# Supplementary material for: Effects of BCG vaccination on donor unrestricted T cells in two prospective cohort studies
Source: eBioMedicine. 2022 Feb 8;76:103839. doi: 10.1016/j.ebiom.2022.103839 (PMC8842032; doi:10.1016/j.ebiom.2022.103839)
Supplement: Supplementary file 1 [file mmc1.docx]

**Supplementary Material**

**Effects of BCG vaccination on donor unrestricted T cells in two prospective cohort studies**

Anele Gela, Melissa Murphy, Miguel Rodo, Kate Hadley, Willem A. Hanekom, W. Henry Boom, John L. Johnson, Daniel F. Hoft, Simone A. Joosten, Tom H.M. Ottenhoff, Sara Suliman, D. Branch Moody, David M. Lewinsohn, Mark Hatherill, Chetan Seshadri, Elisa Nemes, and Thomas J. Scriba, and the Delayed BCG Study Team.

**Supplemental Table S1. Antibody and tetramer panel 1.**

| **Antibody** | **Flourochrome** | **Clone** | **Supplier** |
| --- | --- | --- | --- |
| CD3 | AF700 | UCHT1 | Biolegend |
| CD4 | BV510 | RPA-T4 | Biolegend |
| CD8 | BV785 | SK1 | Biolegend |
| CD26 | BV605 | L272 | BD |
| CD161 | BV650 | DX12 | BD |
| TRAV1-2 | PE-Cy7 | 3C10 | Biolegend |
| CD45RA | PerCP-eFluor 710 | HI100 | Biolegend |
| CCR7 | PE-CF594 | 3D12 | BD |
| HLA-DR | PE-Cy5 | L243 | Biolegend |
| γδ TCR | BV711 | B1 | BD |
| CD1b-GMM | PE |  |  |
| CD1d-PBS57 | APC |  | NIH Tetramer facility |
| MR1-5-OP-RU | BV421 |  | NIH Tetramer facility |
| CD1b/d, and MR1-6FP control tetramers | AF488 |  | NIH Tetramer facility |
| CD14 | APC-H7 | MΦP9 | BD |
| CD19 | APC-H7 | SJ25-C1 | BD |
| Live/Dead | Near-IR |  | Thermofischer |

**Supplemental Table S2. Antibody panel 2.**

| **Antibody** | **Flourochrome** | **Clone** | **Supplier** |
| --- | --- | --- | --- |
| CD16 | BV510 | 3G8 | Biolegend |
| CD3 | APC-H7 | SK7 | BD |
| CD4 | ECD | SFCI1274011 | Beckman Coulter |
| CD8 | BV650 | RPA-T8 | BD |
| CD26 | APC | BA5b | BioLegend |
| CD161 | PE-Cy5 | DX12 | BD |
| TRAV1-2 | BV605 | 3C10 | Biolegend |
| IFN*-*γ | AF700 | B27 | BD |

**Supplemental Table S3. Antibody panel 3.**

| **Antibody** | **Flourochrome** | **Clone** | **Supplier** |
| --- | --- | --- | --- |
| CD33 | BV650 | VM53 | BD |
| CD16 | PE-Cy5 | 3G8 | BD |
| CD3 | APC-H7 | SK7 | BD |
| CD4 | BV786 | SK3 | BD |
| TCR-γδ | BV421 | B1 | BioLegend |
| CD20 | BV711 | 2H7 | BioLegend |
| HLADR | BV605 | L243 | Biolegend |
| IFN-γ | AF700 | B27 | BD |

**Supplementary Figure 1. Power calculations.** Statistical power when comparing two groups of 25 samples each using the Mann-Whitney test for various standardized effect sizes, underlying statistical distributions and statistical significance thresholds (a). We assumed no confounders. Note that the significance thresholds of 0.01 and 0.005 correspond to significance thresholds of 0.05 when the family-wise type I error rate is controlled using the Bonferroni procedure for five and ten hypotheses in a family, respectively. Null (black) and alternate (orange to red) distribution for the normal, beta and gamma distributions for various standardized effect sizes (b).

**Supplementary Figure 2. Longitudinal T cell memory profiles**. T cell memory profiles, measured by CCR7 and CD45RA co-expression patterns, on MR1-5-OP-RU tetramer^+^ MAIT cells (a), CD1d-PBS57 tetramer^+^ NKT cells (b), γδ T cells (c) or total CD4 T cells (d) in BCG-revaccinated adults. Relative proportions of cells that fall into each of the possible combinations of CCR7 and CD45RA are represented as percentages. Circles represent medians at the indicated time points, connected by lines. Error bars represent the IQR. The black dots represent the estimated median. Error bars represent 95% confidence intervals. The p-values are likelihood ratio tests for a difference in mean at any time point. Q-values are Bonferroni-adjusted p-values, with q < 0.05 considered statistically significant.
